# ECG Biometrics via Dual-Level Features with Collaborative Embedding and Dimensional Attention Weight Learning

**DOI:** 10.3390/s25175343

**Published:** 2025-08-28

**Authors:** Kuikui Wang, Na Wang

**Affiliations:** 1 School of Computer Science and Technology, Shandong Jianzhu University, Jinan 250101, China; 2 School of Software, Shandong University, Jinan 250101, China

**Keywords:** dual-level features, dimensional attention weight learning, collective matrix factorization, ECG

## Abstract

In recent years, electrocardiogram (ECG) biometrics has received extensive attention and achieved a series of exciting results. In order to achieve optimal ECG biometric recognition, it is crucial to effectively process the original ECG signals. However, most existing methods only focus on extracting features from one-dimensional time series, limiting the discriminability of individual identification to some extent. To overcome this limitation, we propose a novel framework that integrates dual-level features, i.e., 1D (time series) and 2D (relative position matrix) representations, through collaborative embedding, dimensional attention weight learning, and projection matrix learning. Specifically, we leverage collective matrix factorization to learn the shared latent representations by embedding dual-level features to fully mine these two kinds of features and preserve as much information as possible. To further enhance the discrimination of learned representations, we preserve the diverse information for different dimensions of the latent representations by means of dimensional attention weight learning. In addition, the learned projection matrix simultaneously facilitates the integration of dual-level features and enables the transformation of out-of-sample queries into the discriminative latent representation space. Furthermore, we propose an effective and efficient optimization algorithm to minimize the overall objective loss. To evaluate the effectiveness of our learned latent representations, we conducted experiments on two benchmark datasets, and our experimental results show that our method can outperform state-of-the-art methods.

## 1. Introduction

Identity security is a broad issue, and conventional techniques based on passwords and ID cards are not sufficient for a digital society. Recently, ECG biometrics has gained great momentum as a new solution for human recognition, and it has shown excellent results in existing studies [1]. Specifically, ECG data has many superior characteristics compared with other biometric modalities such as the face, fingerprint, and iris, making ECGs an appropriate means for human identification [2]. For instance, an ECG captures the unique traits of a subject, and all living humans have their own ECG. Meanwhile, ECGs can be continually and non-invasively recorded.

Based on how existing ECG biometric methods extract the features from ECG signals, we can roughly divide them into two categories: fiducial methods and non-fiducial methods. Fiducial methods usually extract the amplitudes, peak ratios, and time intervals between the fiducial points [3,4,5], while non-fiducial methods are obtained from signal transformations, such as discrete cosine and wavelet-based transforms [6,7], statistical features [8,9], and deep learning [10,11]. Due to the success of multi-feature learning in biometric recognition applications, it is generally adopted as a more robust approach to achieve better performance, with some promising results having been reported. For example, Huang et al. [12] present a multi-view discriminant analysis approach in the consideration of sample diversity for ECG biometric recognition. Zhang et al. [13] propose low-rank and joint sparse representations for multi-modal recognition. Wang et al. [14] apply nonnegative matrix factorization with multi-view data in the shared space. Huang et al. [15] propose a unified sparse representation learning framework using multiple features for ECG biometric recognition and they also propose a multi-feature learning framework [16] by extracting three kinds of feature, i.e., 1D-LBP, AC/DCT, and wavelet LBP histograms, to improve recognition performance.

Although existing methods have shown promising results, they may fail to adequately extract information from the original ECG signals. Usually, they treat ECG signals as one-dimensional (1D) and extract the corresponding base features. However, such designs may not be adequate, because 1D signals are inherently limited in complexity, restricting the amount of discriminative information that can be derived from them. Fortunately, some methods treat 1D signals as figures and use image techniques to obtain the base features [17,18]. Inspired by this, we try to represent signals in a two-dimensional (2D) form to obtain more information and integrate these two levels of signals, i.e., 1D and 2D forms, to extract more discriminative features. Therefore, in this paper, we focus on the joint dual-level features of ECG biometrics and explore two significant issues. The first issue is how to maintain the unique characteristics of two-level features. While each level can provide distinct features, it is more advantageous to jointly learn level-specific features and ensure their compatibility through simultaneous modeling. Consequently, the second issue is how to effectively establish correlations between the representations from these two-level dimensions.

**Figure 1:**
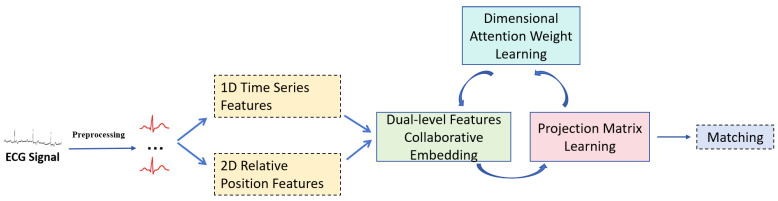
The framework of our method.

To tackle the above issues, we propose a novel framework for ECG biometrics via dual-level features with collaborative embedding and dimensional attention weight learning. The framework of our method is shown in Figure 1. As illustrated in Figure 1, our framework first segments ECG signal into discrete heartbeats, then extracts base features. Specially, we leverage not only the 1D but also the 2D features to form the base features. Thus, these two kinds of features can be fully utilized and as much information as possible can be preserved. Then, we use collective matrix factorization to learn the shared latent representations. To further enhance the representational capacity, we not only add constraints on the latent representations but also use different dimension weights. Finally, we design an effective and efficient optimization algorithm to minimize the overall loss function. Extensive experiments conducted on two widely used datasets demonstrate the effectiveness of our proposed method.

The main contributions of this paper are summarized as follows:

- We propose a novel framework to effectively learn the discriminative latent representation space for ECG biometrics. Our framework mainly has three parts: dual-level feature collaborative embedding, dimensional attention weight learning, and projection learning.
- To solve the overall objective loss, we propose an effective and efficient algorithm for optimization.

The rest of this paper is organized as follows: Section 2 provides a brief overview of related works. Section 3 presents the proposed framework to learn discriminative latent representations for ECG biometrics and Section 4 describes the details of the experimental setup and results. Finally, Section 5 concludes this paper.

## 2. Related Works

As a physiological signal produced by the heart’s electromechanical activity, a typical ECG heartbeat consists of P waves, QRS complexes, and T waves, with their characteristic peaks serving as fiducial points for feature extraction. As previously stated, early ECG biometric recognition primarily concentrates on fiducial-based methods and non-fiducial-based methods, depending on the type of features they utilize [18]. Since fiducial-based methods primarily exploit morphological characteristics of ECG signals, these methods focus on detecting and analyzing fiducial points along with their morphological characteristics, such as amplitudes, angles, durations, intervals, slopes, and so on. The features of fiducial-based methods typically include, but are not limited to, the following: the precise detection of fiducial points, the extraction of temporal features (RR intervals and QT durations), the measurement of amplitude characteristics (P wave amplitude and ST segment elevation), and the calculation of derived parameters (QRS angles and T wave slopes) [5]. For example, refs. [19,20] carefully selected fiducial features, i.e., amplitudes, durations, intervals, and standard deviations of individual wave components, to comprehensively develop the ECG identification system.

Non-fiducial methods offer an alternative solution by avoiding the detection of fiducial points, which effectively improves the discriminability of ECG features and, consequently, enhances recognition performance. Generally speaking, non-fiducial methods can be categorized into three types, including whole-signal analysis techniques, sliding-window-based processing, and transform-domain feature extraction. For example, Sorvillo et al. [6] exploit non-fiducial features by relying on the morphology of the tracing, i.e., cepstal coefficients, mean crossing rate, and scattering transform coefficients. Pinto et al. [7] extract discrete cosine transform and Haar transform features and feed them to decision methods based on support vector machines, k-nearest neighbours, multilayer perceptrons, and Gaussian mixture model and universal background model classifiers for both identification and authentication tasks. Fatimah et al. [21] employ the phase transform and Fourier decomposition to extract ECG features.

In current ECG biometrics, matrix factorization has also achieved remarkable advancements. Numerous studies employ matrix factorization approaches to reduce high-dimensional ECG signals to low-rank latent representations, effectively minimizing the negative impacts of noise interference and information redundancy on identification performance. For example, Huang et al. [16] propose a robust multi-feature collective non-negative matrix factorization model to handle noise and sample variation in ECG biometrics. Wang et al. [22] adopt collective matrix factorization to seek a latent implicit feature space by making full use of the supervised information. Li et al. [23] utilize graph-regularized non-negative matrix factorization to encode the geometrical information and label information to obtain more discriminative features.

Furthermore, to address the limitations of a single modality, adopting a multimodal-based approach serves as a viable alternative for enhancing recognition performance. Boumbarov et al. [24] investigated a multi-modal biometric system that relies on the fusion of facial features and ECG signals. Hammad et al. [25] proposed a secure multi-modal biometric system utilizing various levels of fusion between ECG and fingerprint data, while Bashar et al. [26] explored the fusion of ECG and EEG signals for human identification and El-Rahiem et al. [27] introduced a multi-modal biometric authentication system based on the deep fusion of ECG and finger vein patterns.

Recently, deep learning techniques have received a great deal of attention and have proven to be extremely powerful tools for ECG biometrics [28,29,30,31]. For instance, Abdeldayem et al. [32] firstly segmented the ECG signal and utilized its cyclostationarity and spectral correlation to enrich the signal’s original informational content. They then fed the spectral correlation images into two convolutional neural network (CNN) architectures to determined the final optimized architecture. Rincon-Melchor et al. [33] introduced a deep learning framework employing a Transformer architecture for ECG biometric identification utilizing attention mechanisms. Hazratifard et al. [34] introduced an ensemble siamese network model for ECG signal authentication by employing a CNN model to process the input signals and a comparison component to compare the corresponding extracted features with the claimed sample vectors in the repository to calculate their similarity. Zehir et al. [35] utilized empirical mode decomposition (EMD) to decompose the ECG signals into multiple IMFs and evaluated them by means of two deep learning models: gated recurrent units (GRUs) and long short-term memory (LSTM). While deep learning methods are convenient and sometimes effective, they also have several drawbacks, such as requiring large amounts of training data and incurring high computational costs during training.

## 3. Methodology

### 3.1. Problem Definition and Notation

Generally, ECG biometrics aims to achieve identity recognition with the raw ECG signals as inputs. However, the performance may be unsatisfactory if we directly use these signals to conduct the recognition task. To obtain satisfactory recognition accuracy, researchers have devoted significant efforts to processing ECG signals, extracting effective features, and finding the most discriminative characteristics. Thus, the problem in this paper can be defined as follows: (1) After collecting ECG signals, we first extract their features. (2) Then, our model is trained to find a discriminative latent space where identity recognition can be effectively performed. (3) Considering that query samples are not seen during model training, we need to provide a projection matrix which transforms query samples from extracted features into the learned latent space. (4) Finally, in the latent space, we can match a query sample with a specific subject.

For clear reference, we present some important notations here. Since the feature extraction part is not the key focus of our paper, we use existing techniques to obtain 1D and 2D time-series features from ECG signals (details of feature extraction are presented in Section 4.1). In the literature, using existing ECG features as inputs to build novel models is a conventional practice. Assuming that the training set contains *n* samples, we can use $X_{1}\in R^{d1\times n}$ and $X_{2}\in R^{d2\times n}$ to denote the 1D and 2D features, respectively. Here, d1 and d2 are the corresponding dimensions. Since these *n* samples may belong to *c* different subjects, we define the label matrix $L\in {\left\{0,1\right\}}^{n\times c}$ to describe the relationships among samples and subjects. Specifically, $L_{ij}=1$ means that the *i*-th sample belongs to the *j*-th subject, and $L_{ij}=0$ indicates that the *i*-th sample is not related to the *j*-th subject. Then, both extracted features and labels are used as the inputs of our model.

Some other notations used in this paper are as follows. ${\left\|\cdot \right\|}_{F}$ represents the Frobenius norm and Tr(·) denotes the trace operation. I is an identity matrix. 1 and 0 represent an all-one matrix and an all-zero matrix, respectively.

### 3.2. Dual-Level Feature Collaborative Embedding

In contrast to most existing methods, which only take one kind of feature into consideration, our model absorbs both 1D and 2D time-series features. Our scheme has some clear advantages: First, abundant and diverse data are fed into our model. Second, transformation from ECG signals to 2D features can be viewed as a data augmentation method by offering redundant features of the time series to improve the generalization ability. For the 1D time-series features $X_{1}$, we use the amplitude of the ECG signal to represent the morphological features. For the 2D features, we employ a relative position matrix to convert the raw time series ECG data into 2D features by calculating the relative positions between two timestamps of the time series. This transformation preserves both morphological patterns and temporal dynamics while maintaining strong robustness against signal variations. Then, both 1D and 2D features are fed into the framework to learn discriminative latent representations of subjects. Since collective matrix factorization is used, we further reshape the 2D features into $X_{2}$ to align with $X_{1}$.

Now, the problem is how to fully use these two features. As stated above, we aim to find a discriminative latent space where biometric recognition can be effectively performed. In this space, all samples should have their own latent representations $V\in R^{r\times n}$, where *r* is the dimension of the latent space. Thus, our problem becomes how to learn V from $X_{1}$ and $X_{2}$. The following equation is provided:

(1) $$\begin{array}{c} \min_{U_{1},U_{2},V}\alpha {∥X_{1}-U_{1}V∥}_{F}^{2}+\beta {∥X_{2}-U_{2}V∥}_{F}^{2}\\ \;\;\;\;\;\;\;\;\;\;\;\;\;\;\;\;s.t.\;{\mathrm{VV}}^{⊤}=nI_{r},{V1}_{n}=0_{r}, \end{array}$$

where $U_{1}$ and $U_{2}$ are projection matrices for 1D/2D features and α and β are trade-off parameters.

The above equation collaboratively embeds the extracted features into the latent space, and there is some ingenuity behind this design. (1) We establish direct links between the latent space and extracted features. As a result, V can inherit the abundant and diverse information of 1D and 2D features. (2) With the adopted collective matrix factorization technique, our model can better understand the intrinsic structure of both $X_{1}$ and $X_{2}$ and learn better representations V. By capturing the implicit relationships between different views of ECG data, the generalization ability of the model can be enhanced. (3) To make the to-be-learned space more discriminative, we further add two constraints on V. The first constraint ${\mathrm{VV}}^{⊤}=nI_{r}$ ensures that one dimension is independent of other dimensions. The second one ${V1}_{n}=0_{r}$ is the dimension balance constraint. With these two constraints, we can obtain as much information as possible while enhancing the discriminative power of V.

### 3.3. Dimensional Attention Weight Learning

We defined the latent space by learning the representations V of the training data. However, different dimensions of the latent representations may embody diverse information, with varying levels of significance among them. Therefore, the importance attributed to each dimension differs. To better adapt our learned latent space to our task, we need to incorporate dimensional attention. By revisiting the problem definition, we can easily find that one subject may have several samples, and the representations of one subject should be similar.

To address the above two issues, we propose the following equation:

(2) $$\min_{A,V}{∥S-V^{⊤}AV∥}_{F}^{2}$$

where S is the pairwise similarity among all samples and A is the dimensional attention weight matrix. To tackle the first issue, the diagonal matrix A is added to the inner product of latent representations. By learning this matrix, our model can ensure that different dimensions characterize different aspects and naturally have varying levels of importance. To solve the second issue, we introduce the pairwise similarity matrix S, which is constructed from the label matrix. If two samples belong to the same subject, the corresponding element in S should be 1, and their representations can be guided to be as similar as possible. Furthermore, considering that the size of S is n×n, we let $S=G^{⊤}G$, where G is a two-norm column normalized label matrix with its *j*-th column defined as $G_{*j}=L_{*j}/∥L_{*j}∥$. This design helps our framework avoid square complexity, making the whole procedure efficient.

### 3.4. Projection Matrix Learning

After obtaining good representations V, the latent space can also be determined simultaneously. However, V only contains the seen training samples while query data are not involved. Therefore, we need to establish a mapping from the extracted features to the learned latent space to handle out-of-sample query data. With this in mind, the following formulation can be provided:

(3) $$\min_{W_{1},W_{2},V}\eta {∥W_{1}X_{1}-V∥}_{F}^{2}+\gamma {∥W_{2}X_{2}-V∥}_{F}^{2},$$

where η and γ are trade-off parameters.

### 3.5. Overall Objective Loss

By combining (1), (2) and (3), the overall objective function can be given as follows:

(4) $$\begin{array}{c} \min_{U_{1},U_{2},W_{1},W_{2},V}\alpha {∥X_{1}-U_{1}V∥}_{F}^{2}+\beta {∥X_{2}-U_{2}V∥}_{F}^{2}+\eta {∥W_{1}X_{1}-V∥}_{F}^{2}+\gamma {∥W_{2}X_{2}-V∥}_{F}^{2}\\ \;\;\;\;\;\;\;\;\;\;\;\;\;\;\;\;+{∥S-V^{⊤}AV∥}_{F}^{2}+\zeta Re\left(U_{1},U_{2},W_{1},W_{2}\right),\;\;\;s.t.{\mathrm{VV}}^{⊤}=nI_{r},{V1}_{n}=0_{r}. \end{array}$$

### 3.6. Optimization

To solve the objective loss in (4), we propose an iterative alternating optimization algorithm. Our algorithm consists of several optimization iterations. Within each iteration, there are six sub-problems, where we learn these six variables one by one. More specifically, we optimize one variable with others fixed. The details of one iteration are as follows, and the objective function can converge by repeating several iterations.

**$U_{1}$ Sub-Problem.** Directly and simultaneously learning all variables is challenging because this problem is non-convex. Therefore, we adopt the iterative alternating optimization strategy. Within each iteration, we first optimize the $U_{1}$ sub-problem by fixing all variables except $U_{1}$. Then, (4) can be rewritten as

(5) $$\begin{array}{c} \min_{U_{1}}\;\;\alpha {∥X_{1}-U_{1}V∥}_{F}^{2}+\zeta {∥U_{1}∥}_{F}^{2}. \end{array}$$

The above equation is convex and can be solved easily. We first set the derivative of (5) with respect to $U_{1}$ to zero and then obtain the closed-form solution:

(6) $$\begin{array}{c} U_{1}=\alpha X_{1}V^{⊤}{\left(\alpha VV^{⊤}+\zeta I\right)}^{-1} \end{array}$$

**$U_{2}$ Sub-Problem.** By fixing other variables and omitting terms unrelated to $U_{2}$, the sub-problem is as follows:

(7) $$\begin{array}{c} \min_{U_{2}}\;\;\beta {∥X_{2}-U_{1}V∥}_{F}^{2}+\zeta {∥U_{2}∥}_{F}^{2}. \end{array}$$

We can find that this sub-problem is the same as the $U_{1}$ sub-problem. Hence, we also set the derivative of (7) with regard to $U_{2}$ to zero to obtain its solution:

(8) $$\begin{array}{c} U_{2}=\beta X_{2}V^{⊤}{\left(\beta VV^{⊤}+\zeta I\right)}^{-1}. \end{array}$$

**A Sub-Problem.** First, we reformulate (4) into the A sub-problem:

(9) $$\begin{array}{c} \min_{A}\;\;{∥S-V^{⊤}AV∥}_{F}^{2}. \end{array}$$

Then, the dimensional attention weights are

(10) $$\begin{array}{c} A=diag\left(\left(VV^{⊤}\right)⊙{\left(VV^{⊤}\right)}^{-1}\right)diag\left(VSV^{⊤}\right), \end{array}$$

where ⊙ denotes an element-wise multiplication. A is a diagonal matrix with the weights of each dimension assigned to its diagonal elements.

**$W_{1}$ Sub-Problem.** By fixing other variables and omitting irrelevant terms, the current sub-problem is

(11) $$\min_{W_{1}}\;\;\eta {∥W_{1}X_{1}-V∥}_{F}^{2}+\zeta {∥W_{1}∥}_{F}^{2}.$$

Similar to $U_{1}$ and $U_{2}$, we let the derivative of the above equation be 0 and can obtain

(12) $$\begin{array}{c} W_{1}=\eta VX_{1}^{⊤}{\left(\eta X_{1}X_{1}^{⊤}+\zeta I\right)}^{-1}. \end{array}$$

**$W_{2}$ Sub-Problem.** Consistent with the above sub-problem, we first obtain the problem of $W_{2}$:

(13) $$\min_{W_{2}}\;\;\gamma {∥W_{2}X_{2}-V∥}_{F}^{2}+\zeta {∥W_{2}∥}_{F}^{2}.$$

Then, its closed-form solution is

(14) $$\begin{array}{c} W_{2}=\gamma VX_{2}^{⊤}{\left(\gamma X_{2}X_{2}^{⊤}+\zeta I\right)}^{-1}. \end{array}$$

**V Sub-Problem.** By keeping the other variables unchanged, the objective function to solve the latent representation V can be rewritten as

(15) $$\begin{array}{c} \min_{V}\;\;\alpha {∥X_{1}-U_{1}V∥}_{F}^{2}+\beta {∥X_{2}-U_{2}V∥}_{F}^{2}\\ +\eta {∥W_{1}X_{1}-V∥}_{F}^{2}+\gamma {∥W_{2}X_{2}-V∥}_{F}^{2}\\ +{∥S-V^{⊤}AV∥}_{F}^{2}\;\;s.t.\;{\mathrm{VV}}^{⊤}=nI_{r},{V1}_{n}=0_{r}. \end{array}$$

In contrast to the former problems, we cannot directly optimize V by setting the derivative to zero due to its constraints. To solve it, we first transform (15) into a matrix trace form, and it can be rewritten as

(16) $$\begin{array}{c} \max_{V}\;\;Tr(\alpha U_{1}^{⊤}X_{1}+\beta U_{2}^{⊤}X_{2}+\eta W_{1}X_{1}+\\ \;\;\;\;\gamma W_{2}X_{2})V^{⊤}),\;s.t.\;{\mathrm{VV}}^{⊤}=nI_{r},{V1}_{n}=0_{r}. \end{array}$$

The first constraint ${\mathrm{VV}}^{⊤}=nI_{r}$ ensures that one dimension is independent of other dimensions. The second one ${V1}_{n}=0_{r}$ is the dimension balance constraint. With these two constraints, we can obtain as much information as possible while enhancing the discriminative power of V. Then, according to [36], an analytical solution to (16) can be obtained with the aid of a centering matrix ${J=I}_{n}-\frac{1}{n}1_{n}1_{n}^{⊤}$. Additionally, we define $Z=\alpha U_{1}^{⊤}X_{1}+\beta U_{2}^{⊤}X_{2}+\eta W_{1}X_{1}+\gamma W_{2}X_{2}$. Thereafter, to find a solution of V, we first perform the eigendecomposition of ${\mathrm{ZJZ}}^{⊤}$ as follows:

(17) $$\begin{array}{c} {\mathrm{ZJZ}}^{⊤}=\left[Q\;\;\tilde{Q}\right]\left[\begin{array}{cc} \Omega & 0\\ 0 & 0 \end{array}\right]{\left[Q\;\;\tilde{Q}\right]}^{⊤}, \end{array}$$

where $Q\in R^{r\times r^{\prime }}$ is the corresponding eigenvectors, and $\tilde{Q}\in R^{r\times (r-r^{\prime })}$ is the matrix of the remaining $r-r^{\prime }$ eigenvectors, corresponding to zero eigenvalues. $\Omega \in R^{r^{\prime }\times r^{\prime }}$ is the diagonal matrix of positive eigenvalues, and $r^{\prime }$ is the rank of ${\mathrm{ZJZ}}^{⊤}$. Then, we can obtain an orthogonal matrix $\bar{Q}\in R^{r\times (r-r^{\prime })}$ by conducting a Gram–Schmidt process on $\tilde{Q}$. Furthermore, we define ${U={\mathrm{JZ}}^{⊤}Q\Omega }^{-1/2}$ and a random orthogonal matrix $\bar{U}\in R^{n\times (r-r^{\prime })}$. If $r^{\prime }=r$, $\bar{U}$, $\bar{Q}$, and $\tilde{Q}$ are empty. Ultimately, the optimal solution for V sub-problem is obtained as follows:

(18) $$V=\sqrt{n}\left[Q,\bar{Q}\right]{\left[U,\bar{U}\right]}^{⊤}.$$

**Overall Algorithm.** Our algorithm contains several iterations to be converged and each iteration is constituted of the above six sub-problems. For a more clear understanding of the whole optimization process, we summarize all necessary information in Algorithm 1.

**Algorithm 1** The proposed optimization algorithm**Input**: dual-level features $X_{1}$ and $X_{2}$; L; parameters α, β, η, γ, ζ, and the total iteration number *T*.
**Output**: projection matrices $W_{1}$ and $W_{2}$.
**Main Algorithm**:
  Randomly initialize variables.
  **while** not converged or not reaching the max iterations **do**
   Learn $U_{1}$ sub-problem with (6).
   Learn $U_{2}$ sub-problem with (8).
   Learn A sub-problem with (10).
   Learn $W_{1}$ sub-problem with (12).
   Learn $W_{2}$ sub-problem with (14).
   Learn V sub-problem with (18).
  **end while**


### 3.7. Complexity Analysis

To further comprehend our method, here, we analyse the computational complexity of our method. First, we give the explanations of variations: $d_{1}$ and $d_{2}$ are the original 1D and 2D base feature dimensionality, *c* is number of subjects, *n* is the size of the training set, and *r* is the latent representation dimensionality. Specifically, the complexity of the representation learning step is composed of $O(d_{1}rn+r^{2}n+r^{3}+d_{1}r^{2})$ for solving Equation (6), $O(d_{2}rn+r^{2}n+r^{3}+d_{2}r^{2})$ for solving Equation (8), $O(r^{2}+r^{2}c+rcn+r^{2}n)$ for solving Equation (10), $O(rd_{1}n+{d_{1}}^{2}n+{d_{1}}^{3}+r{d_{1}}^{2})$ for solving Equation (12), $O(rd_{2}n+{d_{2}}^{2}n+{d_{2}}^{3}+r{d_{2}}^{2})$ for solving Equation (14), and $O(crn+2rn+r^{3}+r^{2}+r^{2}n)$ for solving Equation (18), respectively. Since *c*, $d_{1}$, $d_{2}$, *r* ≪ *n*, the overall complexity of training is linear to the size of training set *n* and it is scalable to large-scale datasets.

### 3.8. Matching Process

After the training procedure, the projection matrices $W_{1}$ and $W_{2}$ can be learned for both 1D and 2D time-series features. If query samples and enroll samples are denoted as ${X_{*}}^{query}$ and ${X_{*}}^{enroll}$ (where ∗∈{1,2} represents 1D or 2D features), we can construct their discriminative representations as ${W_{1}X_{1}}^{query}$, ${W_{2}X_{2}}^{query}$, ${W_{1}X_{1}}^{enroll}$, and ${W_{2}X_{2}}^{enroll}$. With the obtained representations, we can calculate the Euclidean distance among the query and enroll samples. If the distance between query *i* and enroll *j* is the smallest for both domains, we can conclude that the query sample *i* is considered to belong to the *j*-th subject.

## 4. Experiments

### 4.1. Experimental Settings

**Datasets.** We conducted the experiments on two datasets—MIT-BIH and PTB—to evaluate the effectiveness of our method. **MIT-BIH** [37,38] is one of the most commonly used datasets for ECG biometrics. The ECG signals for this dataset were collected by multiple electrodes attached on the body and were acquired from 47 individuals. The second dataset, **PTB** [39], includes 549 recordings from 290 subjects. The ECG signals for PTB were collected using 12 conventional leads and three Frank leads. In this paper, we chose 273 subjects and every subject utilized a single lead (I).

**Evaluation Metrics.** To evaluate the performance of our method, we employed the accuracy and equal error rate (EER) as evaluation metrics, where accuracy represents the percentage of correctly identified individual heartbeat signals and EER is a point where the false acceptance rate (FAR) and false rejection rate (FRR) are equal.

#### Signal Preprocessing and Dual-Level Features Extraction

As stated in the above, our method takes the base features $X_{1}$ and $X_{2}$ as inputs of the framework. Before we extracted the base features, we first processed the ECG signals to segment the heartbeats. To achieve this goal, we applied the Pan–Tompkins algorithm [40] to detect the R peak, and selected a fixed number of sampling points from each side of the R peak to form one heartbeat. Each heartbeat in the MIT-BIH dataset contains 260 sampling points, while each heartbeat in the PTB database contains 460 sampling points. For both datasets, we randomly selected 60% of heartbeats per subject as the training data to train the framework, 30% for enrolling, and 10% for probing. Next, for the 1D time-series features $X_{1}$, we used the amplitude of ECG signals to capture the morphological characteristics. The dimensionality of 1D time-series feature is 260 for MIT-BIH and 460 for PTB. For the 2D time features $X_{2}$, we first employed a relative position matrix to convert the raw ECG time series into 2D features by calculating the relative positions between timestamps. Since collective matrix factorization is used, we further reshape the 2D feature into $X_{2}$ to align with $X_{1}$. The dimensionality of 2D features is 676 for MIT-BIH and 1521 for PTB. Both 1D and 2D features were fed into the proposed framework to learn discriminative latent representations of subjects.

### 4.2. Comparisons with State-of-the-Art Methods

We compared our method with several state-of-the-art baselines on MIT-BIH and PTB, and the experimental results are shown in Table 1 and Table 2, respectively. For MIT-BIH, we compare the proposed method with non-deep methods [22,41,42] and deep methods [32,35]. For PTB, we compare the proposed method with non-deep methods [43,44] and deep methods [32,34,35].

Based on these results in Table 1 and Table 2, we make the following observations: (1) Compared with non-deep methods, our method achieves optimal accuracy and EER on MIT-BIH and PTB, demonstrating its superior effectiveness. (2) Compared with deep methods, our method significantly outperforms existing deep approaches in accuracy, while maintaining competitive EER performance. Notably, although the deep methods [32,35] obtain a marginally better EER than our method, our framework’s accuracy improvement is more satisfying. (3) We also explicitly compare the subject counts across state-of-the-art methods. Notably, while [35] reports a superior EER on PTB, this result was obtained using a significantly smaller number of subjects. One possible explanation for these results is that the dual-level features used by our method can capture more detailed information, thereby enhancing the discriminability of the learned representation. The results also indicate that the proposed dual-level feature collaborative embedding with a dimensional attention weight learning mechanism provides representative samples for each subject. From the above analysis, we can conclude that our method is effective and has satisfactory performance.

### 4.3. Ablation Experiments

To validate the effectiveness of each component of our method, we conducted experiments using several variants of our framework for the purposes of an ablation study. The comparison results are summarized in Table 3 and the analyses are detailed as follows.

Influence of dual-level feature collaborative embedding: To eliminate the contribution of dual-level feature collaborative embedding, we designed a variant that takes a single-level feature as the input of the framework. Specifically, we leveraged only 1D and only 2D base features as inputs, designated as CE-Ablation-1D and CE-Ablation-2D, respectively. As shown in Table 3, we can observe that our method achieves significant improvements compared to using only 1D or only 2D features. This demonstrates that our method with dual-level feature collaborative embedding can preserve richer and more diverse information to enhance the generalization ability of our framework.

Influence of dimensional attention weight learning: To verify the influence of dimensional attention weight learning, we designed the variant without dimensional attention weight learning, named DA-Ablation. The experimental results in Table 3 show that the proposed method outperforms DA-Ablation. This situation indicates that dimensional attention weight learning can enhance the discriminability of latent representation dimensions, which is beneficial to improving the recognition performance.

Influence of projection matrix learning: To evaluate the effectiveness of projection matrix learning, we designed the variant without adopting a projection matrix, named PA-Ablation. It can be observed in Table 3 that our method exhibits better performance than PA-Ablation, confirming its critical contribution in latent representation learning.

In summary, the ablation experimental results confirm that our proposed dual-level learning framework is effective and the representations generated by the proposed framework have better discriminability.

### 4.4. Parameter Sensitivity

**Figure 2:**
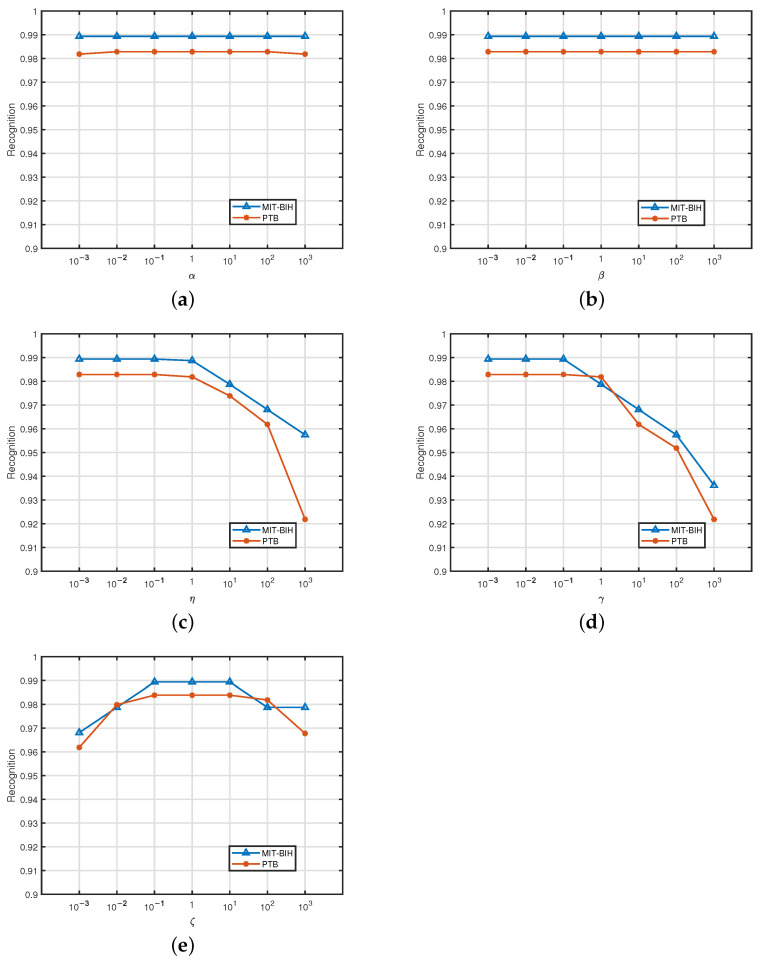
(**a**–**e**) Parameter-sensitive experiments on MIT-BIH and PTB.

We first conducted experiments to analyze the parameter sensitivity in MIT-BIH. There are several main parameters for our method: (1) α and β are the weights of the 1D and 2D feature matrix factorization; (2) η and γ control the projection from base features to the latent representations; and (3) ζ is the trade-off parameter for the regularization term. We let all parameters vary in the range of [0.001,1000] and plot the experimental results in Figure 2. It can be seen that the performance is robust to the different values of α, β, and ζ. The accuracy remains satisfactory when η and γ range from 0.001 to 0.1. Thus, we set α, β, and ζ to 1, and the values of η and γ are all set to 0.01. From the above analysis, we can conclude that our proposed framework is easy to tune in practice.

### 4.5. Comparison with Multi-Feature Biometrics Methods

In order to further validate the effectiveness of our method, we compared our method with multi-feature biometrics methods and the experimental results are outlined in Table 4. Ref. [13] presents a low-rank and joint sparse representation for multi-modal recognition. Ref. [14] applies nonnegative matrix factorization by the $\ell_{21}$ norm with multi-view data in the shared space. From Table 4, we can see that our method outperforms other multi-feature methods on two datasets, validating the effectiveness of our framework.

### 4.6. Convergence Analysis

**Figure 3:**
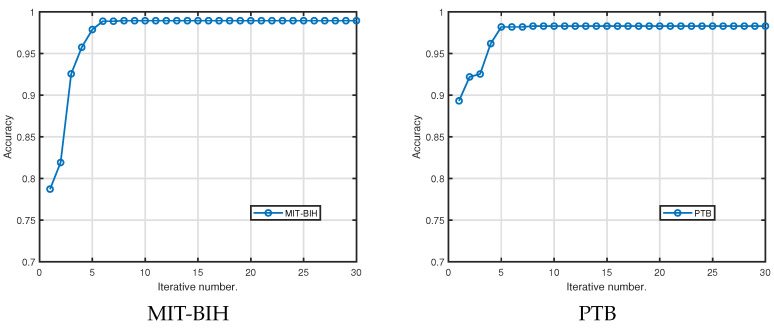
Convergence experiments on MIT-BIH and PTB.

To validate the convergence of our method, we conducted further experiments on two datasets and present the results in Figure 3. Figure 3 depicts the performance trends against the number of iterations. Notably, as the iteration count rises, the accuracy improves steadily within only a few iterations, demonstrating the convergence capability of our method.

### 4.7. Time–Cost Analysis

In this section, we evaluate the computational efficiency of our method on the MIT-BIH by measuring the average time in training, preprocessing, feature extraction, and matching per heartbeat. As summarized in Table 5, the results demonstrate that our method achieves faster processing speeds compared to baseline method, confirming its superior efficiency for real-time ECG biometric applications.

### 4.8. Further Analysis

**Figure 4:**
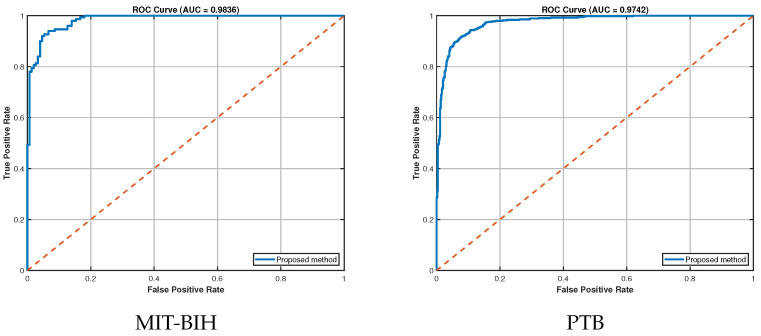
ROC-AUC curve for MIT-BIH and PTB.

To enable more comprehensive evaluation, we introduce additional computational metrics to assess the performance of our method. Specifically, we employ the receiver operating characteristic (ROC) and area under the curve (AUC) as evaluation metrics. As demonstrated in Figure 4, our proposed method achieves strong discriminative performance on MIT-BIH and PTB, with the AUC-ROC visualization confirming its effectiveness.

## 5. Conclusions

In this paper, we propose a new framework for ECG biometrics. We use dual-level features as the input to provide abundant information. Our method contains three main parts, i.e., dual-level feature collaborative embedding, dimensional attention weight learning, and projection learning. Based on the overall objective loss and the proposed optimization algorithm, we can learn the discriminative latent representation space. Experiments were conducted on both datasets and the results have shown that the proposed method is effective.

## Figures and Tables

**Table 1:** Comparison with state-of-the-art methods on MIT-BIH.

Dataset	Method	Number of Subjects	Accuracy (%)	EER (%)
MIT-BIH	[41]	30	96.67	4.57
	[42]	47	93.1	5.78
	[22]	47	94.68	2.73
	[32]	47	96.5	0.3
	[35]	47	98.57	0.73
	ours	47	98.94	0.87

**Table 2:** Comparison with state-of-the-art methods on PTB.

Dataset	Method	Number of Subjects	Accuracy (%)	EER (%)
PTB	[43]	100	97.1	2.88
	[44]	10	97.5	4.58
	[32]	290	94.9	0.25
	[34]	290	96.8	1.69
	[35]	52	98.26	0.93
	ours	273	98.29	1.36

**Table 3:** Results of ablation experiments.

Variant	MIT-BIH	PTB
CE-Ablation-1D	89.36%	86.08%
CE-Ablation-2D	84.50%	79.60%
DA-Ablation	86.13%	81.35%
PA-Ablation	93.62%	92.19%
Our method	98.94%	98.29%

**Table 4:** Comparison with multi-feature biometrics methods.

Dataset	Method	Accuracy (%)
MIT-BIH	[13]	96.87
	[14]	96.32
	OURS	98.94
PTB	[13]	96.72
	[14]	95.68
	OURS	98.29

**Table 5:** Comparison of computational time (seconds) on the MIT-BIH dataset.

Method	Training	Preprocessing	Feature Extraction	Matching
[16]	0.143	0.001	0.009	0.004
ours	0.139	0.001	0.007	0.003

## Data Availability

The original contributions presented in this study are included in the article. Further inquiries can be directed to the corresponding author.
